# Vitality Forms Expressed by Others Modulate Our Own Motor Response: A Kinematic Study

**DOI:** 10.3389/fnhum.2017.00565

**Published:** 2017-11-22

**Authors:** Giuseppe Di Cesare, Elisa De Stefani, Maurizio Gentilucci, Doriana De Marco

**Affiliations:** ^1^Department of Robotics, Brain and Cognitive Sciences, Istituto Italiano di Tecnologia, Genova, Italy; ^2^Neuroscience Unit, Department of Medicine and Surgery, University of Parma, Parma, Italy; ^3^Istituto di Neuroscienze, Consiglio Nazionale delle Ricerche, Parma, Italy

**Keywords:** vitality forms, action style, speech prosody, motor resonance, social interaction, kinematics

## Abstract

During social interaction, actions, and words may be expressed in different ways, for example, gently or rudely. A handshake can be gentle or vigorous and, similarly, tone of voice can be pleasant or rude. These aspects of social communication have been named vitality forms by Daniel Stern. Vitality forms represent how an action is performed and characterize all human interactions. In spite of their importance in social life, to date it is not clear whether the vitality forms expressed by the agent can influence the execution of a subsequent action performed by the receiver. To shed light on this matter, in the present study we carried out a kinematic study aiming to assess whether and how visual and auditory properties of vitality forms expressed by others influenced the motor response of participants. In particular, participants were presented with video-clips showing a male and a female actor performing a “giving request” (give me) or a “taking request” (take it) in visual, auditory, and mixed modalities (visual and auditory). Most importantly, requests were expressed with rude or gentle vitality forms. After the actor's request, participants performed a subsequent action. Results showed that vitality forms expressed by the actors influenced the kinematic parameters of the participants' actions regardless to the modality by which they are conveyed.

## Introduction

Important information about people's behavior is conveyed by the dynamics of the observed action (i.e., the action style). Action dynamics represents an important aspect of the action that has been named “vitality forms” by Stern (2010). Vitality forms are continuously expressed by people and play a dual role in social interactions: namely the execution of vitality forms allows agents to communicate their internal state, while the perception of vitality forms allows receivers to understand the internal states of others (Di Cesare et al., 2015). For example, if an action is performed energetically or gently, one can understand if the agent is angry or calm, or if the agent is performing the action with willingness or hesitancy. The ability to express and to understand the vitality forms is already present in infants (Stern, 1985). These abilities denote a primordial way to relate to and understand others and represent a fundamental constitutive element of interpersonal relations (Trevarthen, 1998; Trevarthen and Aitken, 2001).

Besides the goal (what) and motor intention (why), vitality forms represent a third important aspect of the action: the how. This distinction is not only conceptual, but also anatomical, as has been shown in a previous fMRI study. In particular, Di Cesare et al. (2013) showed that during action observation, paying attention to “what” produced the activation of areas of fronto-parietal “mirror” circuits (Rizzolatti et al., 2014), while paying attention to “how” produced enhanced activation of the right dorso-central insula. In addition, it has been shown that actions performed with different vitality forms are characterized by different kinematic profiles. In particular, physical properties of social actions (i.e., to pass a bottle) performed with rude vitality form have been characterized by a larger trajectory and a higher velocity profile than those performed with gentle vitality form (Di Cesare et al., 2016b). However, despite the importance of vitality forms in social life, vitality forms have been little investigated and it still remains unclear how they could influence our own motor behavior. More specifically, to date, no study has investigated how vitality forms may affect action performance during the response to a social request.

Numerous authors have proposed that action understanding is achieved by a mechanism called motor simulation (Fadiga et al., 1995; Gallese et al., 1996; Rizzolatti et al., 2014), in which an internal replica of the observed action is generated, allowing the observer to simulate the goals or outcomes of the respective action. This assumption implies that, if the motor system is prepared to produce a motor act in response to an action (i.e., social interactive context), this motor performance might automatically replicate some features of the perceived stimulus, showing a “motor contagion” effect (Chartrand and Bargh, 1999; Iacoboni et al., 1999; Heyes, 2011; Bisio et al., 2014). Additionally, a series of studies demonstrated that motor behavior was also sensitive to social context, as for example when participants were asked to interact with a partner expressing a cooperative or competitive attitude (Becchio et al., 2008, 2012; Manera et al., 2011; De Stefani et al., 2015). However, all of these studies investigated the effect of different social intentions (i.e., to cooperate or to compete) in a congruent or incongruent motor task. It still remains unclear how the action style (i.e., vitality form) modulates *per se* the motor behavior during a response to a social request. For this purpose, in the present study we investigate how a specific action request performed by others with different vitality forms (rude and gentle) affected the kinematics of a subsequent motor response of the receiver. For this purpose, participants were presented with video clips showing two actors (a male or a female) performing a giving request (i.e., asking for a bottle) or a taking request (i.e., handing a bottle) presented as visual actions (visual modality) or spoken action verbs (auditory modality) or both (mixed modality). Requests were expressed with rude or gentle vitality forms. During social interactions, vitality forms can be expressed in different modalities, as demonstrated by previous studies in which vitality forms were also conveyed through prosody variation during word or sentence utterance (see De Stefani et al., 2016; Di Cesare et al., 2016a). After the actor's request (visual, auditory, or mixed), participants performed a subsequent action (i.e., a reach-to-grasp a bottle with the goal to give or to take it). Spatial (trajectory) and temporal (velocity and acceleration) features of the participants' motor sequences were measured.

The present study aims to characterize how the receiver's motor action is affected by: (a) the low-level properties (i.e., kinematic profile; see Bisio et al., 2014) of rude and gentle vitality forms; (b) the different goals of the perceived request gestures (i.e., to give or to take possession of an object); (c) modalities of stimulus presentation (visual, auditory, or mixed).

We hypothesized a main effect of vitality forms on the kinematics of the action executed by participants, independently of the modality type. Specifically, we expected a larger trajectory and higher velocity in response to rude vitality forms compared to gentle vitality forms. Moreover, in line with previous studies which demonstrated a clear distinction between neural system codings for action goal (“what”) and vitality (“how”), we expected an effect of vitality forms on the participants' kinematics independent of the meaning of the perceived action requests.

## Materials and methods

### Participants

Fourteen right-handed (Oldfield, 1971) volunteers (eight females and six males; mean age: 24.5 years, SD: ±3.0 years) participated in this study.

The sample size was defined on the basis of results of an “a priori” power analysis computed with GPower 3.1 [Parameters: effect size f(U) = 0.4; α err prob = 0.05; power (1-β err prob) = 0.9]. The output of the analysis revealed that a sample size of 14 subjects is sufficient to evidence an interaction effect between the three experimental factors (see below). Moreover, previous studies which investigated similar effects of social action and language perception on reach and grasp kinematics found significant results using a similar sample size (i.e., between 12 and 14 subjects; see De Stefani et al., 2015, 2016). All participants were native Italian speakers and they had normal or corrected-to-normal vision. The study received approval from the local ethics committee (Comitato Etico per Parma) and was conducted according to the principles expressed in the Declaration of Helsinki. The participants provided written informed consent.

### Apparatus, stimuli, and procedure

**Figure 1:**
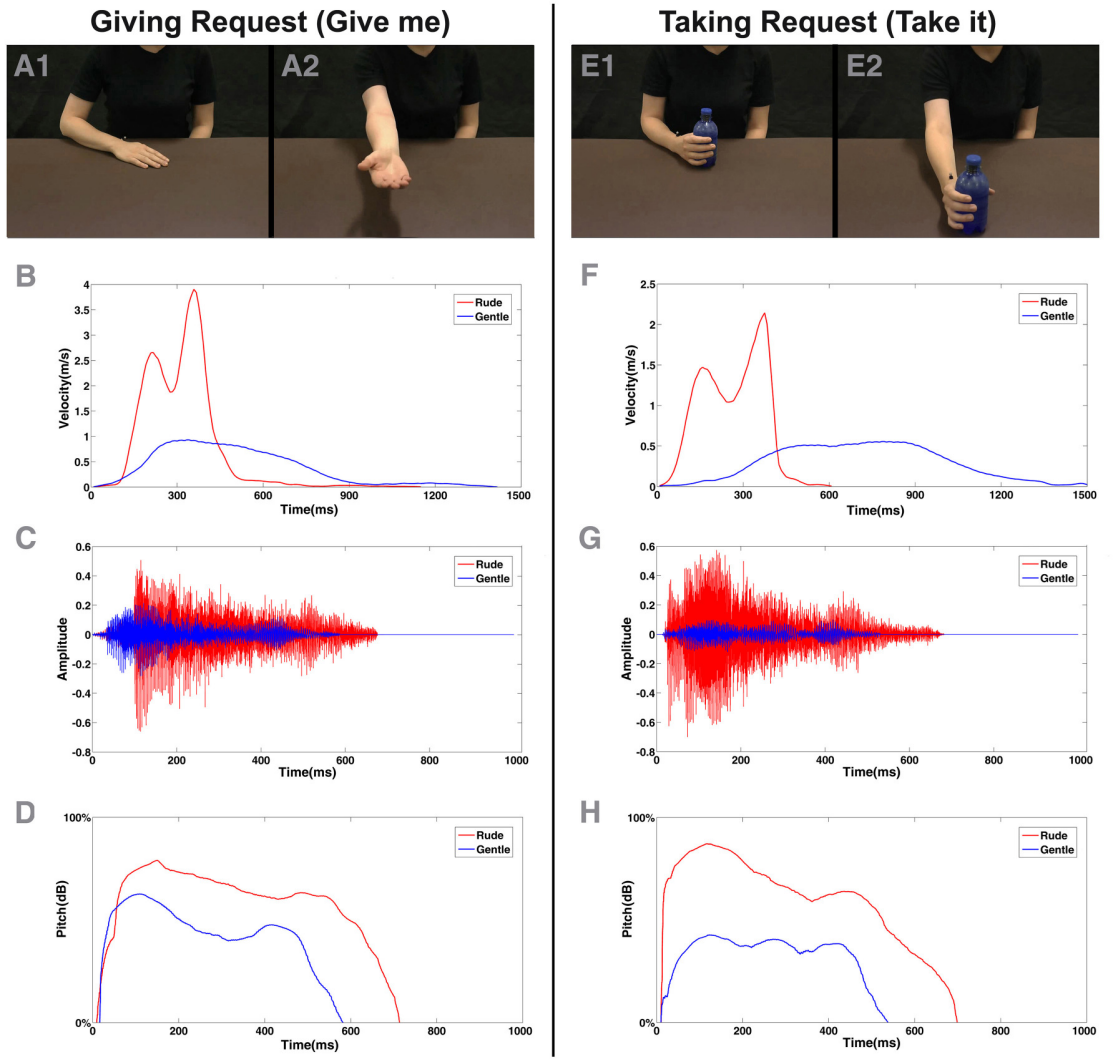
Example of video clips observed by the participants in experiment **(A,E)** and physical properties of stimuli presented in the experiment **(B–H)**. At the top, **(A,E)** depict initial **(A1,E1)** and final posture **(A2,E2)** of the giving and taking requests performed by the actress in visual modality. Under each column **(B,F)**, the plots of physical kinematics computed for each corresponding action were displayed. In the middle, **(C,G)** depict waveform related to rude (red color) and gentle (blue color) action verbs (“dammi” and “prendi”) presented in acoustical modality. At the bottom, **(D,H)** displayed the plots of pitch variation profile of each corresponding verb.

**Figure 2:**
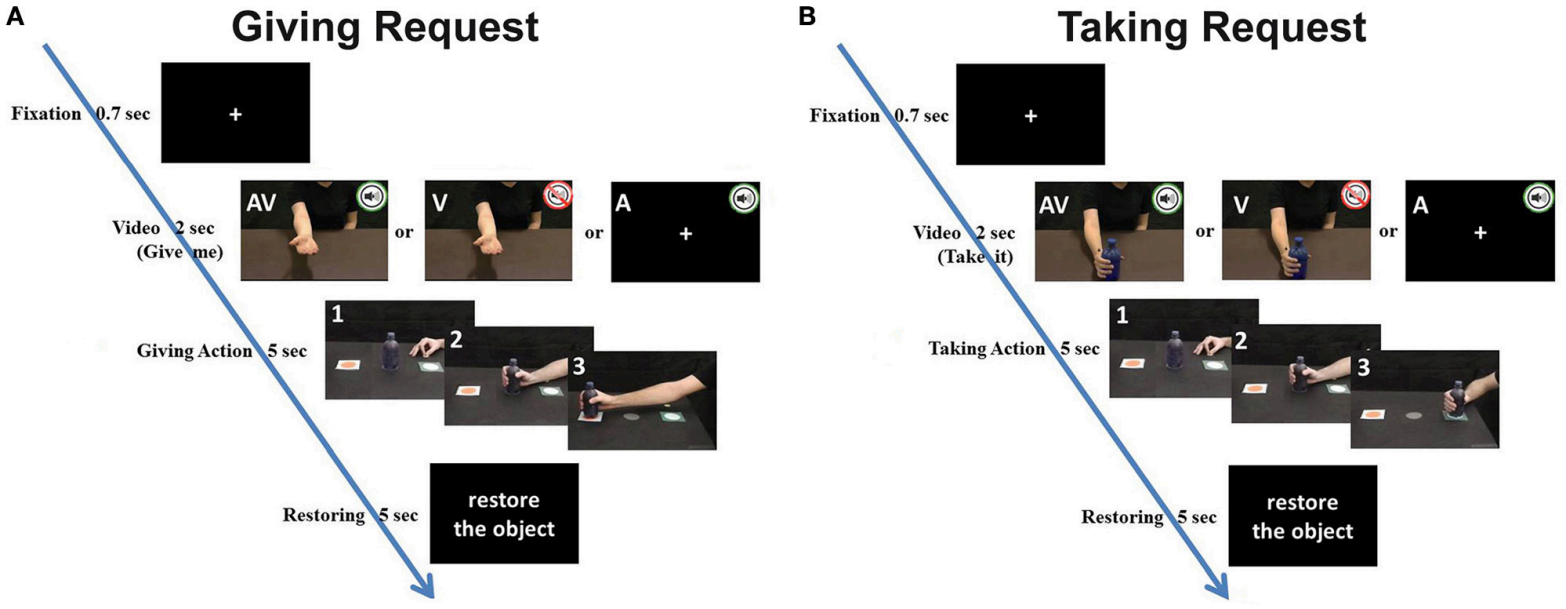
Example of two experimental trials regarding a giving request **(A)** and a taking request **(B)**. Letters in the panels indicate the stimulus modality (A: acoustical stimulus; V: visual stimulus; AV: acoustical and visual stimulus). Panels with numbers displayed the movement phases of participant during each experimental trial: 1, starting position; 2, Grasping the bottle; 3, taking (or giving) the bottle. Time line reports the timing of the different trial phases.

Participants sat comfortably in front of a table, on which they placed their right hand with the thumb and index finger in a pinching position (starting position). The starting position was aligned with the participant's mid-sagittal plane and was 20 cm away from the table edge. The monitor of a computer (19-inch LCD) was placed on the table plane, 70 cm away from the participant's forehead (Figure S1). The monitor was set to a spatial resolution of 1,024 × 768 pixels and at a temporal resolution of 60 Hz. A bottle was positioned on the table 22 cm from the starting position (Figure S1). Stimuli consisted of video clips showing an actor/actress facing the camera and executing two types of requests: a *giving request* (Figure 1A) and a *taking request* (Figure 1E). More specifically, the *giving request* showed actors who: (1) asked for the bottle by moving their right arm toward the participant with the palm upward inviting him/her to give it (visual modality), (2) pronounced the action verb “give me” (auditory modality), (3) both executed the gesture and pronounced the verb simultaneously (mixed modality). The *taking request* showed actors that: (1) placed a bottle in front of participant inviting him/her to take it (visual modality), (2) pronounced the action verb “take it” (auditory modality), (3) both executed the gesture and pronounced the verb simultaneously (mixed modality). The style of the action performed by the actors could be rude or gentle (Figures 1B,F), and similarly, the utterance of the spoken action verb could be pleasant or rude (Figures 1C,G). Stimuli with the same modality were presented in three separate blocks (visual, auditory or mixed modality), counterbalanced between participants. In each block, actions performed with rude or gentle vitality form were presented 10 times each (five trials with a male actor and five with a female actor). In total, 40 stimuli per block were randomly presented (120 stimuli per participant in the whole session). Participants were requested to observe the video clips or to listen to the spoken action verbs and to perform a subsequent action (taking or giving; Figure 2). Each trial started with a fixation cross displayed on a black screen that lasted 700 ms. Then, a video clip showed a visual request (with or without audio) or a verbal request [Italian spoken verbs: “prendi” (take it) or “dammi” (give me) pronounced in imperative mood]. If the stimulus was a *giving request*, participants had to reach for, grasp, and move the bottle close to the monitor (Figure 2A); otherwise, if the stimulus was a *taking request*, participants had to reach, grasp, and move the bottle close to their body (Figure 2B).

### Physical properties of the stimuli

#### Video stimuli

Participants were shown video clips representing two actors, one of whom performed a taking action or a giving action (Figures 1A,B) performed with either gentle or rude vitality forms (eight stimuli in total: 2 actions × 2 actors × 2 vitality forms). The video stimuli were recorded using a high definition camera (Panasonic HCX 900) fixed at a 180° angle with respect to the actors (i.e., providing an opposite point of view). During the action's execution, the kinematics of the actors' arm movements were recorded with the 3D-optoelectronic SMART system (BTS Bioengineering, Milano, Italy). In particular, six video infrared cameras (sampling frequency: 120 Hz) detected the 3D position of a reflecting marker (5-mm-diameter spheres) placed on the wrist of the actors' right hand. The spatial resolution was 0.3 mm. During action execution, the natural, and ecological expression of vitality forms of both actors was preserved as much as possible and the distance between the starting position and the ending position was kept constant. After kinematic recording, the velocity of all eight recorded actions was analyzed using MATLAB (The Mathworks, Natick, MA). Figures 1B,F shows a graphic representation of velocity parameters relative to actions performed by the actress (see also Figure S2 for the male actor's plots). Mean values of peak velocity and maximal amplitude for each arm movement are reported in Table 1. An independent sample *t*-test comparing scores for those exposed to rude vs. gentle actions in order to compare spatial and temporal values in a time-window (328 ms) which included 20 time points located around the maximal values of velocity and trajectory. Results showed that all gentle actions were statistically different from rude actions for movement amplitude and velocity (see Table 1 for statistical values).

**Table 1:** Mean values, Standard Deviations (SD), and significant effects of statistical analysis (paired *t*-tests between gentle and rude stimuli) of kinematic **(A)** and vocal parameters **(B)** relative to visual and auditory stimuli.

**(A) VISUAL STIMULI (REACH PHASE)**							
	**TAKING REQUEST—TAKE IT (“prendi”)**				**GIVING REQUEST—GIVE ME (“dammi”)**		
	**Rude**	**Gentle**			**Rude**	**Gentle**	
**MOVEMENT AMPLITUDE (mm)**							
Mean	7.24	4.69		Mean	12.89	6.78	
SD	4.55	0.59		SD	7.28	0.87	
**MOVEMENT VELOCITY (mm/s)**							
Mean	895.33	566.19		Mean	1,592.67	810.29	
SD	534.39	72.44		SD	852.18	125.73	
**Significant effects (rude vs. gentle)**							
**Measures**	**Effect**	***t*****-value**	***p*****-value**	**Measures**	**Effect**	***t*****-value**	***p*****-value**
Movement amplitude	Rude > Gentle^*^	*t*_(40)_ = 3.9	0.0004	Movement amplitude	Rude > Gentle^*^	*t*_(40)_ = 5.6	0.0001
Movement velocity	Rude > Gentle^*^	*t*_(40)_ = 4.2	0.0001	Movement velocity	Rude > Gentle^*^	*t*_(40)_ = 6.1	0.0001
**(B) AUDITORY STIMULI (SPOKEN ACTION VERBS)**							
	**TAKING REQUEST—TAKE IT (“prendi”)**				**GIVING REQUEST—GIVE ME (“dammi”)**		
	**Rude**	**Gentle**			**Rude**	**Gentle**	
**PITCH VARIATION (Hz)**							
Mean	231.35	198.86		Mean	226.23	208.88	
SD	3.82	7.32		SD	0.68	1.82	
**INTENSITY (dB)**							
Mean	77.60	71.50		Mean	75.32	71.71	
SD	1.51	1.65		SD	2.04	1.55	
**Significant effects (rude vs. gentle)**							
**Measures**	**Effect**	***t*****-value**	***p*****-value**	**Measures**	**Effect**	***t*****-value**	***p*****-value**
Pitch Variation	Rude > Gentle^*^	*t*_(10)_ = 13.5	0.0001	Pitch Variation	Rude > Gentle^*^	*t*_(10)_ = 30.7	0.0001
Intensity	Rude > Gentle^*^	*t*_(10)_ = 14.5	0.0001	Intensity	Rude > Gentle^*^	*t*_(10)_ = 17.6	0.0001

Statistical significance (

* *p < 0.05)*.

#### Audio stimuli

The voice of the actors was measured by using a light-weight dynamic headset microphone (frequency response: 50–15,000 Hz). The microphone was connected to the computer by a sound card (16 PCI Sound Blaster; Creative Technology Ltd., Singapore), and audio was acquired using the Avisoft SAS Lab Professional software (Avisoft Bioacoustics, Germany). The actor's voice parameters (pitch and intensity) were successively measured using the PRAAT software (www.praat.org; PRAAT settings: Pitch, range 75–500 Hz; Analysis method, Autocorrelation; Intensity range, 50–100 dB; Average method, mean energy; Silent threshold, 0.03; Voicing threshold, 0.45). Auditory stimuli were presented by using two loudspeakers (Creative, Inspire T10) connected to the computer. The same analysis used for video stimuli was also carried out for audio stimuli. In particular, we compared the values of pitch variation and intensity along the time window corresponding to the duration of pronunciation of the accented vowel (mean length = 100 ms/11 time points). Figure 1 shows plots of the time-course of pitch (Figures 1D,H) and intensity (Figures 1C,G) values for the female actors' action verbs pronunciation. Male actor's plots are shown in Figure S2. All the action verbs resulted in statistically different differences both for pitch variation and intensity (see Table 1).

#### Testing for subjective stimuli differences: behavioral analysis

Subjective evaluation of visual and auditory stimuli were assessed carrying out a behavioral study on 10 volunteers. In particular, participants were requested to judge each stimulus by using emoticons which expressed anger or kindness. Moreover, in order to avoid an obligatory choice between the positive and negative emotions, the verbal label “don't know” was added as a third possible response. Participants were instructed to observe/listen the stimuli and then to judge them using one of the three possible choices (positive emoticon, negative emoticon, don't know). It is important to note that the classifications “rude” or “gentle” were not mentioned to the participants. Results showed that participants correctly recognized stimuli as rude or gentle with a very high accuracy level [giving request: visual modality (97% rude; 97% gentle; 6% don't know), auditory modality (98,5% rude; 94% gentle; 7,5% don't know), mixed modality (94,5% rude; 94% gentle; 11,5% don't know); taking request: visual modality (97,5% rude; 97,5% gentle; 5% don't know), auditory modality (98,5% rude; 96% gentle; 5,5% don't know), mixed modality (95% rude; 96% gentle; 9% don't know)]. These results clearly demonstrate that participants were able to identify rude or gentle stimuli at subjective level.

### Data recording

Kinematic data of participants were acquired by using the 3D-optoelectronic SMART system (see detailed description above). For each participant two reflective markers were placed on the participants' right thumb and index finger nails (grasping markers). By recording the time course of the distance between the thumb and the index finger, we analyzed the kinematics of the grasping phase. The grasp was constituted by an initial phase of the fingers opening up to a maximum (maximal finger aperture), followed by a phase of the finger closing on the object (Jeannerod, 1988). A third marker was placed on the wrist of each participant in order to analyze the kinematics of the reaching phase (reaching marker).

The kinematic data recordings during the participants' movements were analyzed using MATLAB (R2008b). All parameters were recorded and calculated on three-dimensional axes (X, Y, Z). A Gaussian low-pass smoothing filter (sigma value: 0.93) was applied to the recorded data. The time course of reach-grasp and lift was visually inspected in order to identify the beginning and the end of the entire movement. The beginnings of the reach and grasp phases were defined based on different criteria. The beginning of the grasp was considered to be the first frame in which the distance between the two markers placed on the right finger tips was larger than 0.3 mm with respect to the previous frame and did not decrease under a minimum spatial resolution for at least three consecutive frames. The end of the grasp was the first frame after the beginning of finger closing in which the distance between the two right fingers was smaller than 0.3 mm with respect to the previous frame and did not increase over minimum spatial resolution for at least three consecutive frames. The beginning of the reaching phase, corresponding to the start of movement, was the first frame during which the displacement of the reaching marker along any Cartesian body axis increased with respect to the previous frame and did not decrease under a minimum spatial resolution for at least three consecutive frames. To determine the end of the reaching phase, we calculated separately for the X, Y, and Z axes the first frame following movement onset in which the X, Y, and Z displacements of the reaching marker did not change in comparison with the previous frame. Then, the frame endpoint temporally closer to the grasping end frame was chosen as the end of the reach.

To analyze the arm movements of participants, we measured the following reaching parameters: reach trajectory, reach peak velocity, and reach peak acceleration. These parameters are the indices of the velocity and amplitude of the transport component of the movement. We also analyzed the following grasp parameters: the grasp peak velocity of the fingers (aperture and closure) and the grasp maximal finger aperture (maximal 3D Euclidian distance between the fingers). These grasping parameters were analyzed in order to determine the velocity and amplitude features of the grip phase. We computed these reaching and grasping parameters to assess the effects on the initial and central part of the reach-to-grasp action, which depends on planning and execution control. The selection of these parameters was in accordance with those observed during the stimuli recordings which highlighted the kinematic difference between the rude and gentle actions (see Figure 1 and Table 1). The parameters related to the lift phase were not considered in the analysis because of differences in kinematics execution between a taking and a giving action, making the effects of the style not discernible from the effect of the task.

### Data analysis

A repeated measures MANOVA was carried out for the mean values of the reaching-grasping parameters of the participants (*Reach*: Reach Amplitude, Reach Peak Velocity, Reach Peak Acceleration; *Grasp*: Maximal finger aperture, Grasp Peak velocity of finger aperture, Grasp Peak velocity of finger closure). The within-subject factors were *modality* (visual, auditory, mixed), *action meaning* (taking or giving request), and *vitality form* (rude and gentle). Outlier values were calculated for each subject (>2.5 SD of subject mean) and were discarded from the subsequent statistical analysis (2.3% of the total trials). The significance level was fixed at *p* = 0.05. Sphericity of data was verified before performing statistical analysis (Mauchly's test, *p* > 0.05). All variables were normally distributed (Kolmogorov-Smirnov Test, *p* > 0.05). Effect size was measured by calculating partial η^2^. In accordance with our experimental hypothesis we planned and computed a series of simple contrasts for each single parameter in order to test the differences within *vitality form* condition (rude vs. gentle). No *post-hoc* test was planned considering the absence of any additional significant main or interaction effects in the MANOVA analysis.

## Results

**Figure 3:**
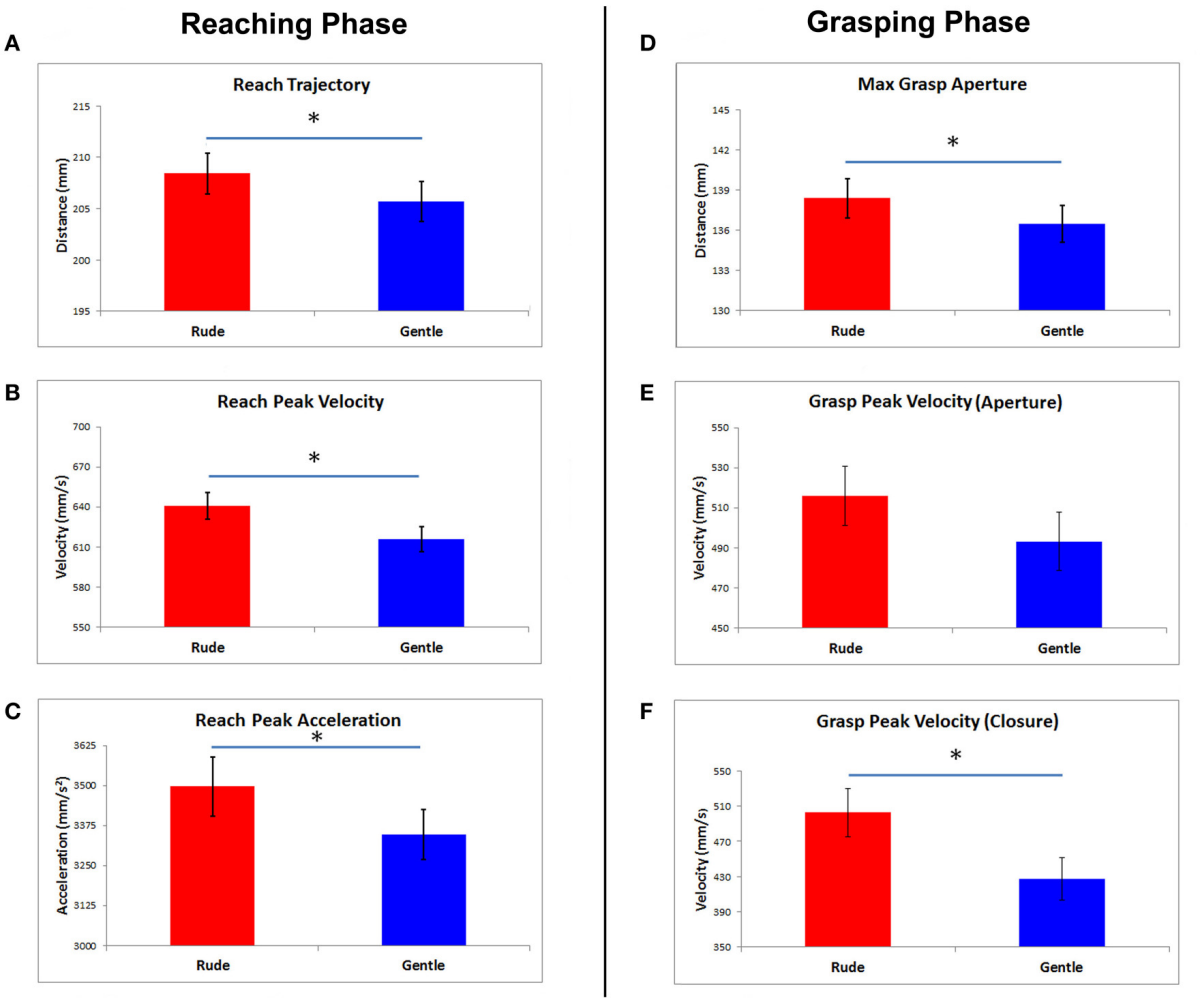
In the left side, the histogram bars display the mean values of reach phase parameters obtained in response to rude and gentle vitality forms **(A–C)**. The mean values of grasp phase parameters are presented in the right side **(D–F)**. Vertical bars represent the standard errors (SE). Horizontal bars indicate statistical significance (^*^p < 0.05).

MANOVA results showed a significant main effect of *vitality form* [Wilks lambda: *F*_(3, 11)_ = 11.5, *p* = 0.001, η^2^ partial = 0.9]. No other significant main or interaction effects were found. Simple contrasts showed that all *Reach* parameters significantly differed for the rude and gentle *vitality forms* (Reach Amplitude *p* = 0.003, Reach Peak Velocity *p* = 0.016, Reach Peak Acceleration 0.03). In particular, the trajectory of the wrist was wider in response to rude vitality form than in response to gentle vitality form, independently from the action meaning or modality (Figure 3A). Additionally, peaks of velocity and acceleration were higher in the rude than in the gentle vitality form condition (Figures 3B,C).

Concerning the *Grasp* phase, the contrast between rude and gentle *vitality form* for Maximal finger aperture and Grasp Peak velocity of finger closure was significant (*p* = 0.008 and *p* = 0.004, respectively; Figure 3F). Grasp peak velocity of finger aperture was very close to significance (*p* = 0.06). The distance between the fingers was significantly wider in response to rude than gentle vitality (Figure 3D), and the finger closure phase of the grasp was faster (Figure 3E). All mean values and SDs are reported in Table 2.

**Table 2:** Mean values and standard deviation (SD) of kinematic parameters relative to Taking and Giving requests during the three different modalities.

	**Visual modality**								**Auditory modality**								**Mixed modality**							
	**Taking request**				**Giving request**				**Taking request**				**Giving request**				**Taking request**				**Giving request**			
	**Rude vitality**		**Gentle vitality**		**Rude vitality**		**Gentle vitality**		**Rude vitality**		**Gentle vitality**		**Rude vitality**		**Gentle vitality**		**Rude vitality**		**Gentle vitality**		**Rude vitality**		**Gentle vitality**	
	**Mean**	**SD**	**Mean**	**SD**	**Mean**	**SD**	**Mean**	**SD**	**Mean**	**SD**	**Mean**	**SD**	**Mean**	**SD**	**Mean**	**SD**	**Mean**	**SD**	**Mean**	**SD**	**Mean**	**SD**	**Mean**	**SD**
Reach trajectory(mm)	209	14	207	14	211	17	207	15	207	13	205	15	208	18	205	16	206	16	205	16	209	16	205	15
Reach peak velocity(mm/s)	648	90	613	69	653	104	608	75	642	64	625	57	635	66	621	62	630	83	618	95	638	84	610	84
Reach peak acceleration(mm/s^2^)	3,586	787	3,338	589	3,580	936	3,281	569	3,508	621	3,420	649	3,442	639	3,408	637	3,401	785	3,325	809	3,465	845	3,313	783
Maximal finger aperture(mm)	139	11	138	11	139	11	136	11	138	12	136	11	137	11	135	11	139	11	137	11	138	10	137	10
Grasp peak velocity of finger aperture(mm/s)	517	136	488	113	494	114	471	108	521	116	494	128	500	134	502	128	544	142	506	119	520	104	498	110
Grasp peak velocity of finger closure(mm/s)	518	193	418	142	514	223	418	170	498	202	433	195	477	229	437	207	500	218	431	201	509	241	428	184

## Discussion

Social interactions are characterized by interpersonal mutual exchange of vitality forms. The expression of vitality forms allows the agent to communicate his or her own internal state while the perception of vitality forms allows the receiver to understand those of others. Understanding vitality forms means to capture the style of an action (i.e., “how” it is performed), rather than its content (i.e., “what” is being done) or the motor intention characterizing it (i.e., “why” it is being done).

The first aim of the present study was to investigate whether and how, during social interaction between participants and an agent (presented by a video-clip), vitality forms expressed by the virtual agent modulate the kinematic parameters of the motor responses of the participants. The second aim was to assess whether the motor responses of participants were also affected by the goals of the request gestures (i.e., to give or to take possession of an object). Finally, we assessed the effect of different modalities (visual, auditory, or mixed) of stimuli presentation on participants' motor responses.

Results indicated that, both for those who witnessed a giving request and those who witnessed a taking request, the perception of vitality forms modulated the kinematic parameters (i.e., velocity and trajectory) of the subsequent actions performed by the participants. Furthermore, participants' responses were not affected by the modality in which agents' requests were conveyed (visual, auditory, or mixed modality). This was valid for both the reach and grasp components of the motor sequences executed by participants. Specifically, vitality forms modulated the temporal (acceleration and velocity) and spatial parameters (trajectory) of the reach component, evidencing larger trajectory, and higher velocity in response to rude requests compared to gentle ones. Additionally, concerning the grasp component, results showed a larger maximal finger aperture in response to rude vitality form than the gentle vitality forms. Furthermore, rude requests speeded up grip closure in the final phase of grasping.

Previous evidence showed how kinematics could be influenced by reciprocal interpersonal perception during an interactive task (Sacheli et al., 2012), showing how the negative valence of interpersonal relation with receivers affected their motor behavior. However, our findings have highlighted the crucial role of vitality forms as an intrinsic feature of action and speech, which modulates the response to a social request independently from the task and other social cues (e.g., facial expression, body posture, etc.). In line with these results, De Stefani et al. (2016) showed a similar effect of emotional prosody on a receiver's motor responses. Specifically, the execution of a feeding motor sequence toward the actress who pronounced the sentence evidenced faster movement in the reach phase in response to positive vs. negative sentences (De Stefani et al., 2016).

It is important to note that the effect of the agent's vitality forms on the motor responses of the receiver also occurred when participants simply listened to spoken action verbs (“dammi,” “prendi”) pronounced with rude or gentle vitality forms. This suggests that the influence of vitality forms on the participants' motor responses cannot merely be ascribed to a mechanism such as, motor imitation. In particular, during vitality forms perception, physical parameters characterizing the action (velocity, trajectory), or the spoken action verbs (pitch, intensity) may be selectively encoded in the dorso-central insula. Indeed, a series of fMRI studies has demonstrated that this insular sector is involved in vitality form processing (Di Cesare et al., 2013, 2015, 2016a,b). Additionally, Di Cesare et al. (2017a) have recently demonstrated that the dorso-central insula is activated not only when participants *observed* or *imagined performing* action vitality forms but also when they *listened to* or *imagined pronouncing* action verbs with gentle and rude vitality forms. These findings clearly indicate that the insular cortex has a role in the processing of multimodal vitality forms, suggesting the existence of a mirror mechanism specific for vitality forms. Unlike the classical fronto-parietal mirror circuit, which plays a role in action goal understanding, this insular mechanism allows one to express their own affective states and to understand those of others. The role of the dorso-central insula would transform the visual/acoustic information into a motor domain, allowing the receiver to understand vitality forms expressed by others and prepare the subsequent motor response.

An important aspect to discuss concerns the role of the perception of each physical parameter in vitality processing. Indeed, it is plausible that the perception of physical parameters characterizing vitality forms (visual modality: velocity, trajectory; auditory modality: pitch, intensity) may have influenced the participant's response. Is it possible to hypothesize that just velocity is responsible for vitality form perception? On the basis of the results of a previous study of Di Cesare et al. (2016b), we can exclude this possibility. In particular, the authors demonstrated a dissociation between velocity and vitality form perception at the behavioral and neural level. More specifically, in this study, participants were presented with video clips showing different social actions (e.g., passing a bottle, a can, or a jar) performed with different velocities (ranging from low to high speed) and were asked to pay attention to and rate either their velocity or their vitality forms. The results showed that, although the stimuli presented in the two tasks were identical, a significant difference was present in the subjects' judgment according to whether they were required to classify the observed actions for their vitality form or their velocity. In addition, fMRI results showed that in the dorso-central insula there were discriminative voxels selectively tuned to vitality forms perception but not to velocity. In addition, another study by Di Cesare et al. (2016a) demonstrated that even for the auditory modality, only the loudness of stimuli cannot account for the perception of vitality forms. Pooling together, these findings suggest that vitality forms are characterized by a combination of different physical parameters which characterize “how” actions and speech are expressed.

In conclusion, our study clearly demonstrated how the perception (observation/listening) of different vitality forms modulates motor behavior in response to a social request. When a conspecific asks us something, his or her positive or negative approach conveyed by the vitality form modulates our subsequent motor response. Our data highlight the fundamental double role of vitality forms during interpersonal interactions. Vitality forms allow us to express our own internal state by shaping our motor output and understanding the output of others. Given their relevance in social communication, it will be important in the future to address the role of vitality forms in social and communicative disorders such as, autism (Rochat et al., 2013; Di Cesare et al., 2017b).

## Author contributions

GD, ED, MG, and DD: designed research; GD, ED, and DD: performed research; GD, ED, and DD: analyzed data; GD, ED, MG, and DD: wrote the paper.

### Conflict of interest statement

The authors declare that the research was conducted in the absence of any commercial or financial relationships that could be construed as a potential conflict of interest.

## Supplementary material

Figure S1 Experimental setting of experiment 1. Blue lines indicate the distances expressed in cm.

Figure S2 Example of video clips observed by the participants in experiment **(A,E)** and physical properties of stimuli presented in the experiment **(B–H)**. At the top, **(A,E)** depict initial **(A1,E1)** and final posture **(A2,E2)** of the giving and taking requests performed by the male actor in visual modality. Under each column **(B, F)**, the plots of physical kinematics computed for each corresponding action were displayed. In the middle, **(C,G)** depict waveform related to rude (red color) and gentle (blue color) action verbs (“dammi” and “prendi”) presented in acoustical modality. At the bottom, **(D, H)** displayed the plots of pitch variation profile of each corresponding verb.
