# Supplementary figures and images for: Vitality Forms Expressed by Others Modulate Our Own Motor Response: A Kinematic Study
Source: Front Hum Neurosci. 2017 Nov 22;11:565. doi: 10.3389/fnhum.2017.00565 (PMC5698685; doi:10.3389/fnhum.2017.00565)

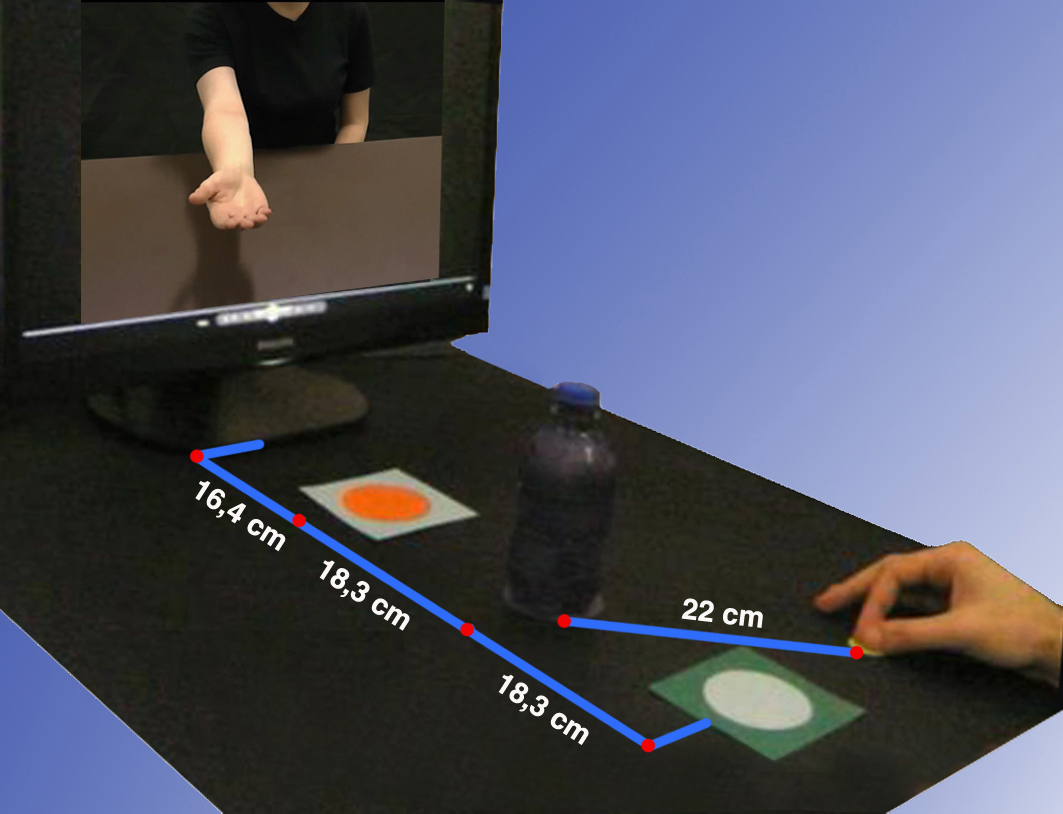

Supplement: Figure S1 — Experimental setting of experiment 1. Blue lines indicate the distances expressed in cm. [file Image1.JPEG]

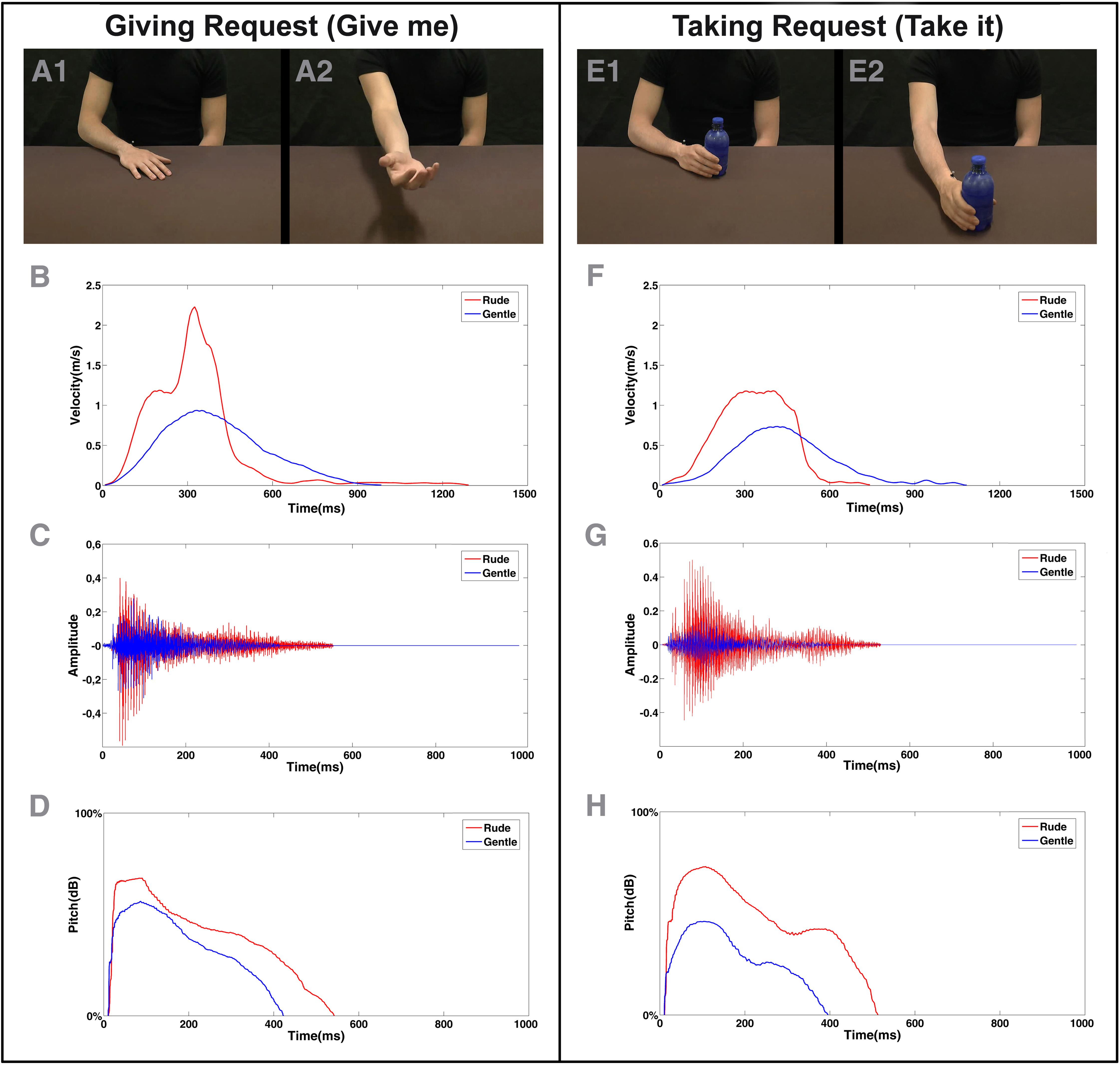

Supplement: Figure S2 — Example of video clips observed by the participants in experiment (A,E) and physical properties of stimuli presented in the experiment (B–H). At the top, (A,E) depict initial (A1,E1) and final posture (A2,E2) of the giving and taking requests performed by the male actor in visual modality. Under each column (B, F), the plots of physical kinematics computed for each corresponding action were displayed. In the middle, (C,G) depict waveform related to rude (red color) and gentle (blue color) action verbs (“dammi” and “prendi”) presented in acoustical modality. At the bottom, (D, H) displayed the plots of pitch variation profile of each corresponding verb. [file Image2.JPEG]
